# A data infrastructure for the assessment of health care performance: lessons from the BRIDGE-health project

**DOI:** 10.1186/s13690-017-0245-1

**Published:** 2018-01-24

**Authors:** Enrique Bernal-Delgado, Francisco Estupiñán-Romero

**Affiliations:** 1 Institute for Health Sciences in Aragon (IACS), Zaragoza, Spain; 2 BRIDGEHealth Project, Brussels, Belgium; 3 Health Services and Policy Research Group - ARiHSP, Institute for Health Sciences in Aragon (IACS), San Juan Bosco 13 (CIBA building), 50009 Zaragoza, Spain

## Abstract

The integration of different administrative data sources from a number of European countries has been shown useful in the assessment of unwarranted variations in health care performance. This essay describes the procedures used to set up a data infrastructure (e.g., data access and exchange, definition of the minimum common wealth of data required, and the development of the relational logic data model) and, the methods to produce trustworthy healthcare performance measurements (e.g., ontologies standardisation and quality assurance analysis). The paper ends providing some hints on how to use these lessons in an eventual European infrastructure on public health research and monitoring. Although the relational data infrastructure developed has been proven accurate, effective to compare health system performance across different countries, and efficient enough to deal with hundred of millions of episodes, the logic data model might not be responsive if the European infrastructure aims at including electronic health records and carrying out multi-cohort multi-intervention comparative effectiveness research. The deployment of a distributed infrastructure based on semantic interoperability, where individual data remain in-country and open-access scripts for data management and analysis travel around the hubs composing the infrastructure, might be a sensible way forward.

**Electronic supplementary material:**

The online version of this article (10.1186/s13690-017-0245-1) contains supplementary material, which is available to authorized users.

## Background

The current EU healthcare agenda is built upon three pillars: strengthening healthcare effectiveness, increasing accessibility and improving resilience. The agenda bestows a critical role, among other strategies, to the assessment of health systems performance and to the routinely use of existing health information systems [1].

There are some countrywide examples where national health institutions have implemented actions meant to use health information systems in the evaluation of health systems performance [2–6]. Although less frequent, there are also some pre-eminent international efforts on cross-country comparisons. Notably, the OECD is regularly producing the Health at a Glance report [7] or numerous outlets from its Healthcare Quality Indicators project [8], and, the European Commission has set up an expert group on health systems performance assessment (HSPA) whose agenda is led for the exchange of HSPA experiences, the definition of HSPA priority areas and the support to national policy-makers on HSPA methods [9]. Lastly, different EU research programs have fostered the development of research initiatives aiming the cross-country analysis of health systems performance. [An extensive review of those research projects can be found at http://www.euroreach.net/compendium]. A commonality between these initiatives is the use (reuse) of routinely collected data, in particular, administrative data.

One of those projects has been ECHO (European Collaboration of Healthcare Optimization) an international effort to access and link administrative health data sources from several European countries with a view to set the basis for cross-country health systems performance assessment. ECHO accessed and reused individual-level data from hospital admissions and, demographic, socioeconomic and supply information to analyse and report on a number of health system performance (HSP) dimensions (e.g., utilisation of low-value procedures, equity of access to effective care, or quality and efficiency), at meaningful levels of analysis (either hospitals or geographic healthcare areas) [10]. (See more details at www.echo-health.eu). Later on, integrated within the context of the BRIDGE-Health project (www.bridge-health.eu), ECHO methods and achievements have been revisited with the aim to contribute to the design and development of a sustainable European infrastructure on public health research and monitoring.

This paper provides a description of the challenges faced to build a data infrastructure aiming HSPA, and some thoughts on whether this model is suitable for a European research infrastructure.

### Challenges on building a health information system based on routine data on health

Any project using routine data to provide sound international HSP research and monitoring has to face a number of challenges; thus: a) defining the minimum common dataset required to assess HSP dimensions and indicators; b) analysing the data origins, as well as the linkage mechanisms and developing the logic data model that will allow the production of comparable performance indicators; c) getting access to original data sources, curated and maintained by data authorities under a predefined legal frame; d) transforming raw data formats and categories into a common standard; e) building extensive catalogues (i.e. dictionaries) aimed to allocate data to units of analysis while considering over time modifications; f) building a common language (i.e., semantic interoperability) from different ontologies (e.g., different diagnoses and procedures classification systems); g) releasing resulting datasets that allow HSP analyses and reporting; and h) analysing the quality of those resulting datasets and, accordingly, decide on the accuracy and reliability of HSP results. In the following paragraphs, some of those critical challenges are discussed taking as a reference the works done in ECHO within BRIDGEHealth (EwB).

#### Definition of the minimum common dataset (MCD)

A project on HSPA aims at reporting relevant dimensions of HSP at meaningful levels of analysis. In the case of international comparisons, once performance dimensions and indicators, as well as units of analysis are decided, an HSPA project has to define the minimum common dataset (MCD) required for the production of such performance indicators.

Operationally, the MCD is the set of variables that composes the so-called ‘core facts’ table of a data infrastructure. The ‘core facts’ table is used to integrate the original administrative data from any participant country into a single coherent relational database. The MCD includes: a) patient attributes (e.g., group of age, sex, diagnoses and procedures); b) episode attributes (e.g., date of admission, type of discharge, type of admission, etc.); c) geographical location (e.g., health care area of residence, health care area of treatment); and, d) hospital of treatment.

Besides, the MCD contains univocal identifiers to grant traceability and linkage across data sources and catalogues: a) univocal identifiers at the maximum level of disaggregation (i.e., episode) and, b) univocal identifiers for the units of analysis, (hospitals, health authorities, or regions). A detail of the MCD from the EwB project is provided in Additional file 1.

#### Data origins, linkage mechanisms and logic data model

The EwB data infrastructure has included various data origins [hospital discharges, demographic information at geographic level, socioeconomic data at geographic level, supply features at hospital level, and geographic vectors depicting geographic areas of interest (e.g., hospital-catchment areas [11], health authorities, regions, NUTS)] with a view to, departing from individual episodes, analysing HSP both, at geographic and hospital-level, accounting for over time evolution (e.g., hospital merging processes).

So, the EwB logic data model, conceived as a relational database, has been built upon three main entities (episodes, hospitals, and geographic areas) and their respective attributes. The critical attributes for each of these entities are described along various catalogues; so, dictionaries or ontologies containing codes for diagnoses and procedures, hospital names, locations and evolution, name for the geographic areas and resident population.

EwB data model owns three critical elements: a) as episodes store individual patient-level information that is actually embedded into both a hospital of treatment and geographic areas hierarchically constituted (health care into regions, regions into countries) linkage across files and catalogues follows either a 1-to-1 scheme (when linkage is limited to episode-based attributes) or a 1-to-N scheme (when episodes are linked to a hospital or an area). The aforementioned MCD univocal identifiers grant traceability and robustness for this linkage; b) given the rich information contained in each episode, mainly dependent on the large number of diagnoses and procedures recorded in each registry (~20 to 50 variables each), there is a need of increasing computational efficiency. For this purpose, the diagnoses and the procedure variables were split into two different catalogues containing all existing diagnoses or procedures, at their maximum level of precision; and, c) instead of yielding a single output, the data model implies the production of three separate output files containing geographic-based HSP indicators, hospital-based HSP indicators, and ad hoc intermediate variables and modifiers to grant the control of confounding (e.g., Elixhauser comorbidities); all are indexed using the EwB univocal identifiers, reducing the volume of files, allowing storage in a distributed way, and enabling parallel access and processing.

Last but not least, as any relational data model the EwB dataset has been tested to check coherence, and whether the model preserves the identity of the entities contained, their referential integrity, the cardinality of the data and, the inheritances of their attributes [12]. A detail of the logic data model is provided in Additional file 2.

#### Standardizing data and assuring semantic interoperability

**Fig. 1:**
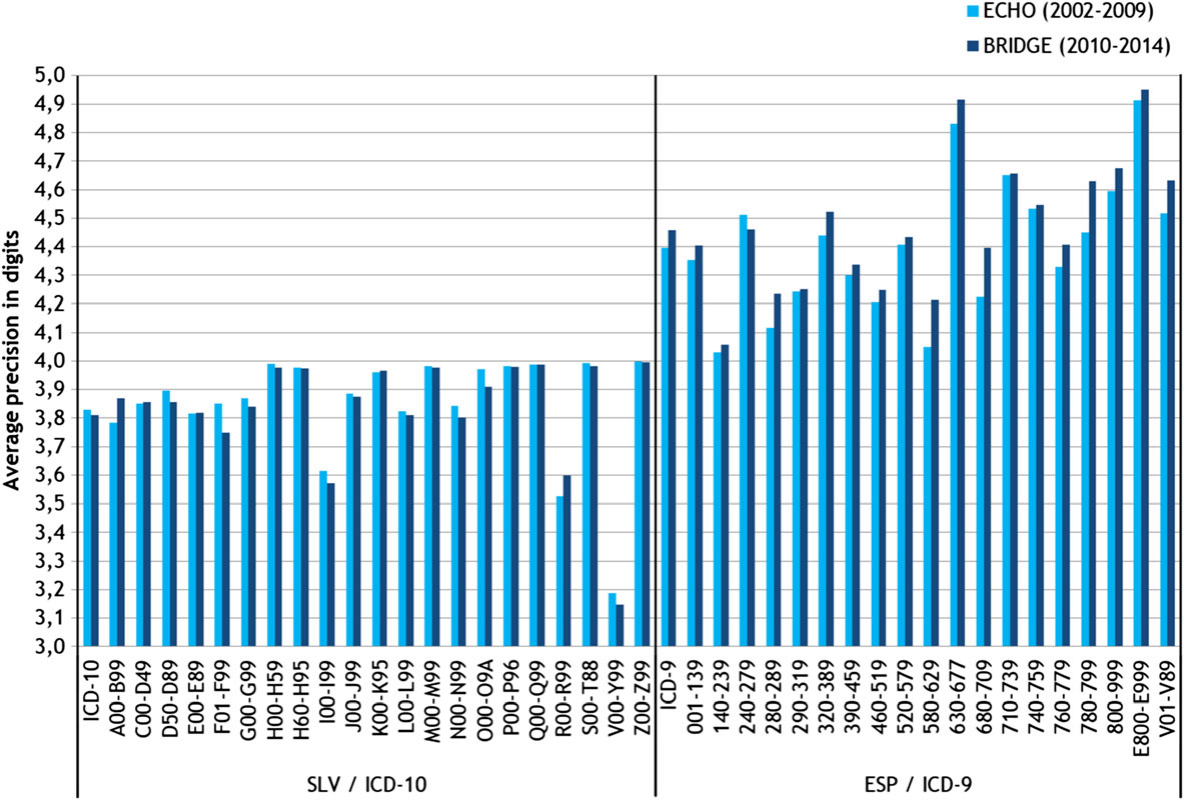
Coding precision (Slovenia and Spain)NNote. Note: Coding precision refers to the level of specificity in the way an admission is coded. In the case of diagnoses or procedures, the different classification systems own different levels of specificity. For example in ICD 9th MC, diagnoses might be coded with three, four of five digits; the greatest level of specificity implies the use of 5 digits. In the figure, actual levels of specificity in Slovenia (ICD 10th) and Spain (ICD 9th) are shown. Spain shows larger coding precision in all Major Diagnostic Categories (x axis), generally improving since 2010. When building comparable indicators the level of specificity has to be equivalent

**Fig. 2:**
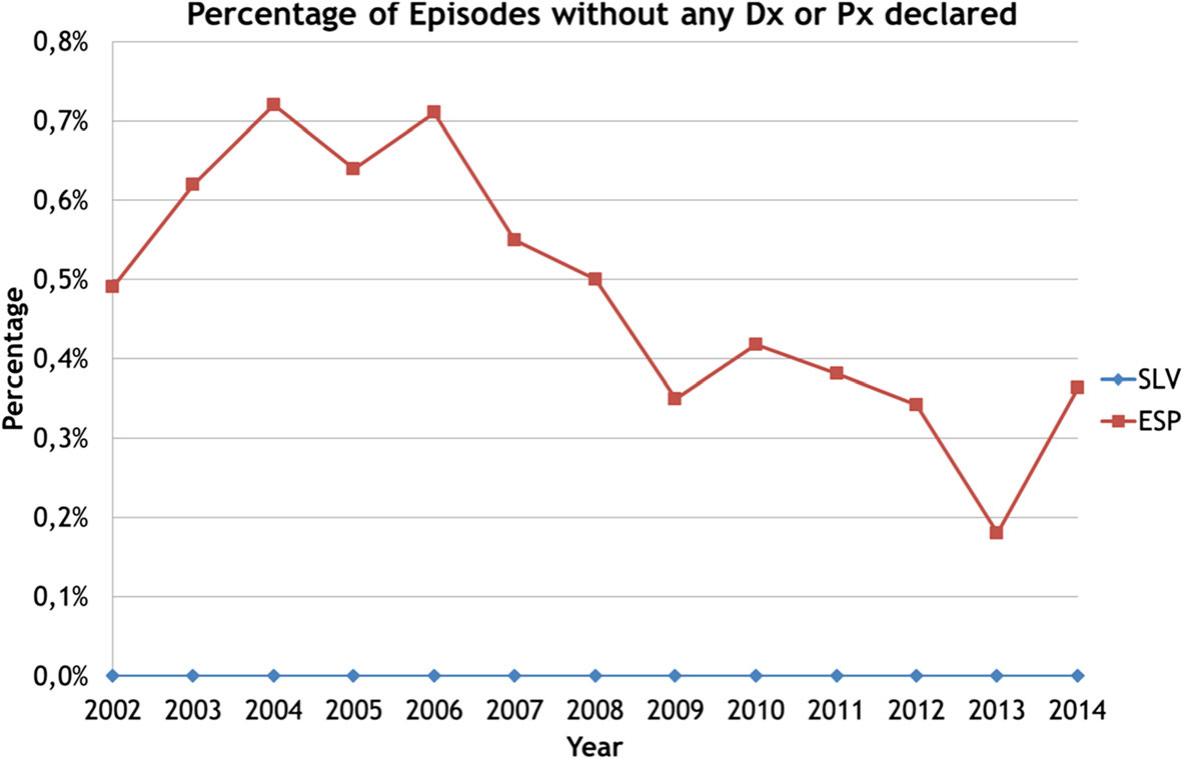
Missing values in core variables. Note: The figure shows the evolution in the number of episodes with missing information on the diagnosis of admission or on the procedures performed in the episode. While Slovenia has no defaulting episodes, in Spain the number has reduced over time ranging from 0.7% to 0.4%. The toll of missing data in either the diagnosis of admission or in the procedures would impact the numerators and/or denominators of the performance rates

**Fig. 3:**
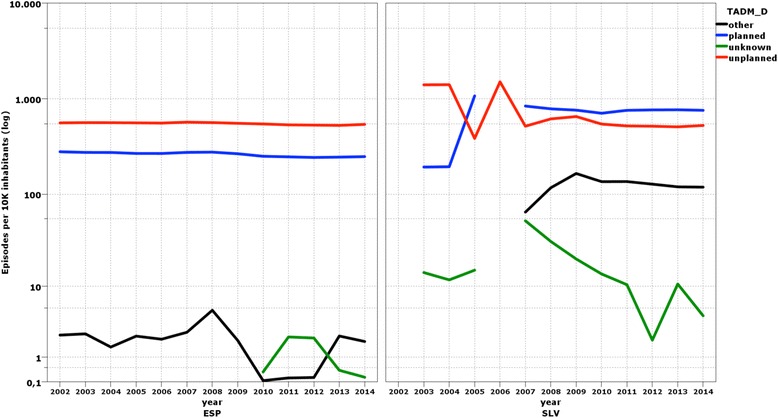
Time-dependent phenomena – episodes by type of admission. Note: The figure on the right aims at highlighting the Slovene inconsistency (as compared to Spain) in the coding of planned (blue line) vs. unplanned admissions (red line), as well as disproportionally large number of ‘other’ types of admission. Apparently, in 2006 all admission were coded as unplanned, and since 2007 a new category ‘other type of admission’ starts to be used. This timedependent finding might have an impact in those performance indicators that require this variable – typically, unplanned admission are excluded when assessing surgical outcomes that might be influenced by differences in acute and severe patients’ conditions

The standardization of data from different data origins and different countries entail a series of operations that aim the comparability of the raw data. In the use of administrative data the most frequent threats to comparability are: a) the way variables are categorized might be different across data origins; b) coding precision maybe different hampering the comparability of definitions (Fig. 1); c) not all data origins might provide full coverage for each of the variables at any unit of analysis (Fig. 2); d) time-dependent phenomena might translate into data inconsistencies over the years (Fig. 3); and, e) data origins might use different ontologies to normalize their data.

The level of complexity of the remedies will differ. The differences in the way the variables are categorized will generally require a simple transformation looking for a minimum common denominator across origins. Differences in coding precision will entail careful assessment to weight the actual impact on the construct validity of the performance indicators. Missing values or time-dependent modifications will involve a logic data model that flexibly deal with both issues; so, in the former creating ‘missing’ categories, at each level of analysis; in the latter, creating comprehensive catalogues linked with secondary identifiers able to account for changes (e.g., in geographic boundaries, the merge of several providers or the periodic upgrade of classification systems).

Nonetheless, the greater level of complexity lays on tackling the semantic differences across ontologies; for example, whether an episode means the same in all the data origins, or whether what a coding system flags as congestive heart failure in an origin, is a congestive heart failure in another one. Semantic interoperability is at the very core of any HSPA international comparison as HSPA culminates with the development and measurement of performance indicators using information on diagnoses and procedures frequently, from different ontologies and coding systems (In EwB, for example, ICD9th, ICD 10th, ACHI, NOMESCO and OPCS).

Addressing semantic interoperability requires indicator-specific crosswalks across coding systems. For that purpose, an extensive mapping of codes has to be developed, which implies a deep knowledge of the different ontologies and the face and empirical validation of in-country experts (coders, clinicians and potential users of HSPA results) looking for coherence, anomalous data distributions and consistency over time. Taking the case of EwB, the mapping exercise has ended up producing crosswalks for 201 indicators. [For more details on the crosswalks see: http://www.echo-health.eu/handbook/getting-indicators.html].

#### Assuring internal validity through data quality analysis

As any observational study, HSPA is threatened by systematic bias and confounding. The use of routine administrative data in observational studies requires further efforts to understand any potential information flaw. It is then essential a systematic analysis of the quality of the dataset upon which HSPA is developed. A concise example on in-hospital case-fatality rates in the admission for an acute myocardial infraction (AMI-IM) is provided to have a hint on how to systematically analyse information flaws in the dataset. For that purpose, data from Slovenia and Spain, a total of 65 million episodes (5 million a year in Spain and half a million a year in Slovenia, from 2002 to 2014) have been analysed and discussed hereinafter.

AMI-IM is operationally defined as the standardised in-hospital case-fatality rate for patients over 18 years old, admitted for an acute myocardial infarction. So, besides the quality analysis of case-fatalities (i.e., numerator) and patients 18 and older admitted for an AMI (i.e., denominator), as HSPA requires risk-adjustment so that hospital differences in performance, beyond differences in the underlying risks in patients, may be controlled. The quality of potential confounders has also to be analysed. (Crosswalks may be consulted at http://www.echo-health.eu/handbook/CV_AMI_MORT.html).

**Fig. 4:**
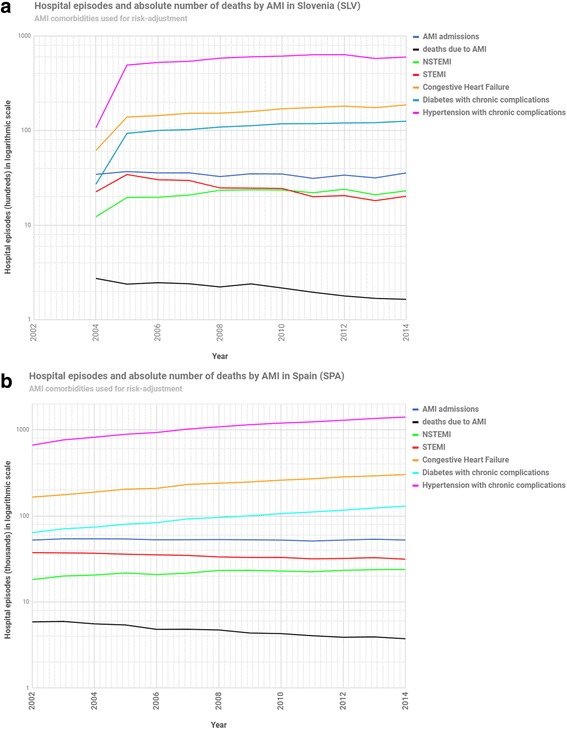
ᅟ

Looking at Fig. 4 a great stability is observed, in both countries, in the main variables concurring in this indicator; thus: a) case-fatalities steadily and smoothly decrease as expected (black line); b) AMI admissions following a similar pattern in both countries (dark blue line), suggesting that no systemic factors are affecting unevenly in the number of patients at risk; and, c) there is consistent evolution of potential confounders (AMI as STEMI or NSTEMI, the concurrence of congestive heart failure, the presence of comorbidities as diabetes with complications and hypertension with complications) with no abrupt changes as those seen in Fig. 2. In general terms, HSPA comparisons with this indicator seem to be accurate. Let’s look though more in depth. In the Slovene figure, stability starts at some point after 2005, so HSPA will be more reliable after that year. Most importantly, in both countries ST-elevation of myocardial infarction (STEMI) is reducing (red line) while Non-STEMI (NSTEMI) episodes (green line) are appearing more frequently –overlapping in Slovenia since 2008 and not yet converging in Spain. It is very unlikely that the AMI epidemiology is changing over the years (i.e., more NSTEMI cases in the latest years). Conversely, it is more plausible to think of a potential information bias; so, besides coding differences between Slovenia and Spain, it seems that in-country coding is very likely underestimating STEMI cases -it is well known that more severe patients that end up dying in the first 24 h (STEMI cases) have less precise information in their discharge records, end up being registered as undefined AMI. As a consequence, the use of STEMI vs. NSTEMI as potential confounders in the risk adjustment of AMI-IM, between Slovenia and Spain, has to be carefully assessed, and maybe discarded.

### Developing HSPA with administrative data in an EU health information infrastructure

EwB has developed a central relational data infrastructure that stores administrative data from different data sources from various countries, with a view to carry out health systems performance research and monitoring. Might this data infrastructure be taken as a reference in the context of an eventual EU health information infrastructure? Along the following paragraphs, we reflect on those elements that should be taken into consideration in the public debate on the development of such an infrastructure at the EU level.

Some of the EwB attainments would suggest that the data infrastructure developed could be taken as a valid case study. Thus,

- EwB has proven the plausibility of creating a central repository populated with anonymised and de-identified individual information, transferred from different countries with limited administrative costs, while attaining the various legal requirements in data access, management, curation and reporting.
- As EwB builds on administrative data (i.e., data regularly collected by health or statistic authorities upon the compliance of normative provisions) it benefits of certain stability over time, irrespective of in-country health care reforms.
- Albeit the uneven richness of data across Europe, EwB has revealed that it is possible to find a minimum common dataset that eventually allows a sound comparison of health systems performance at meaningful units of analysis.
- The logic data model enables individual, hospital-specific and geographic analyses; moreover, it allows the traceability of these episodes, hospitals and geographic areas, capturing time-dependent phenomena that might alter their consistency over time, and subsequently, the reporting of performance.
- A method has been developed to assure semantic interoperability in the development of performance indicators addressing different HSP domains: utilization, equity, quality and safety, and efficiency. As aforementioned, the data model allows straightforward updates once the new ontology is available.
- All methods and techniques are transparent and publicly available with a view to assure reproducibility.

Nonetheless, a translation of the EwB model into a European Research Infrastructure would not be straightforward.

- EwB has been conceived as a demonstration project developed in a limited number of countries, a sample of convenience that, although representative of countries with different data governance schemes and data richness, might not represent the complexity of the EU28.
- EwB is confined though to the secondary use of hospital administrative data (enriched with some extra administrative data sources) aimed at specifically analysing health care performance, which may not be the only type of data sources (nor the only aim) in an eventual European Research Infrastructure. Nevertheless, the challenges addressed along this paper are not specific to hospital data, so should be quite the same for any other data source.
- In practical terms, the administrative costs for the maintenance of an expanded EwB infrastructure is unknown; on the other hand, the legal requirements for data access (and eventual transfer) will multiply, so that the exiguous governance model implemented in EwB might not suffice.
- A third element has to do with the semantic interoperability. Although the method developed to build comparable performance indicators has been shown valid, there is a need of in-country expert panels contributing in the face and empirical validation of existing or new indicators, as well as to the attentive follow up of the publication of renewed ontologies. So, the EwB governance of this task should not be the same when scaling up to more indicators and more countries.
- Last but not least, although the EwB central relational dataset has been proven accurate, effective to compare HSP across different countries, and efficient enough to deal with hundred of millions of episodes, the logic data model might no be responsive to some future requirements. On the one hand, the data model requires individual data transfer; although this has been possible in some countries, others have found serious difficulties ending up not providing data. On the other hand, although this logic data model allows research and monitoring, it is confined to ecological or cross-sectional studies that, at most, may add a temporal perspective.
  According to the current developments in health systems performance beyond classical monitoring, a state-of-the-art infrastructure should aim the reuse of electronic health and medical records and conduct more complex comparative effectiveness research. Should the EU data infrastructure aim at addressing the challenge, it might be recommendable a different type of logic data model for which we propose a distributed model where individual data remain in-country and open-access scripts for data extraction, transformation, analysis and reporting travel around the hubs composing the infrastructure.

## Additional files

Additional file 1: Annex 1. (PDF 85 kb)

Additional file 2: Annex 2. (PDF 260 kb)
