# Supplementary material for: A data infrastructure for the assessment of health care performance: lessons from the BRIDGE-health project
Source: Arch Public Health. 2018 Jan 24;76:6. doi: 10.1186/s13690-017-0245-1 (PMC5784587; doi:10.1186/s13690-017-0245-1)
Supplement: Supplementary file 1 — Annex 1. (PDF 85 kb) [file 13690_2017_245_MOESM1_ESM.pdf]

ANNEX 1 –Minimum Common Dataset

Variable name	Description	Definition	SPAIN	PORTUGAL	SLOVENIA	DENMARK	ENGLAND	AUSTRIA	HUNGARY
rec	clinical record number								
id	patient id								
zip	address post code	address post code							
hospital	hospital id	hospital of treatment							
country	country identification	country identification							
mare1	meaninful area level 1	area of residence - country							
mare2	meaninful area level 2	area of residence - region							
mare3	meaninful area level 3	area of residence - health care area							
mare4	meaninful area level 4	area of residence - municipality							
year	year	year							
age	age	age							
gqe	age group	age group							
echokey	echo key								
bdate	brith date	patient's birth date							
sex	sex	patient's gender							
adate	admission date	patient's date of admission							
tadm	type of admission	patient's admission to the hospital: programmed/urgent/other							
disdate	discharge date	patient's date of discharge							
tdis	type of discharge	type of patient's discharge: alive/dead/tranfered/other							
intdate	intervention date	patient's date of surgery							
diag1	principal diagnosis	patient's principal diagnosis							
diag2-30	secondary diagnoses	patient's secondary diagnoses							
proc1	main procedure	main procedure							
proc2-30	other procedures	secondary procedures							
dcc	day case care	day-case surgery/diagnostic & therapeutic procedures							
mfund	modality of funding	if the stay/procedure was funded by public or private							
los	length of stay	length of stay							
readm	readmission	readmission							

Note: Dark green cells represent the information common to all countries. Yellow cells represent the information lacking in some countries.
