# Supplementary material for: A data infrastructure for the assessment of health care performance: lessons from the BRIDGE-health project
Source: Arch Public Health. 2018 Jan 24;76:6. doi: 10.1186/s13690-017-0245-1 (PMC5784587; doi:10.1186/s13690-017-0245-1)
Supplement: Supplementary file 2 — Annex 2. (PDF 260 kb) [file 13690_2017_245_MOESM2_ESM.pdf]

Annex 2. Logic Data Model

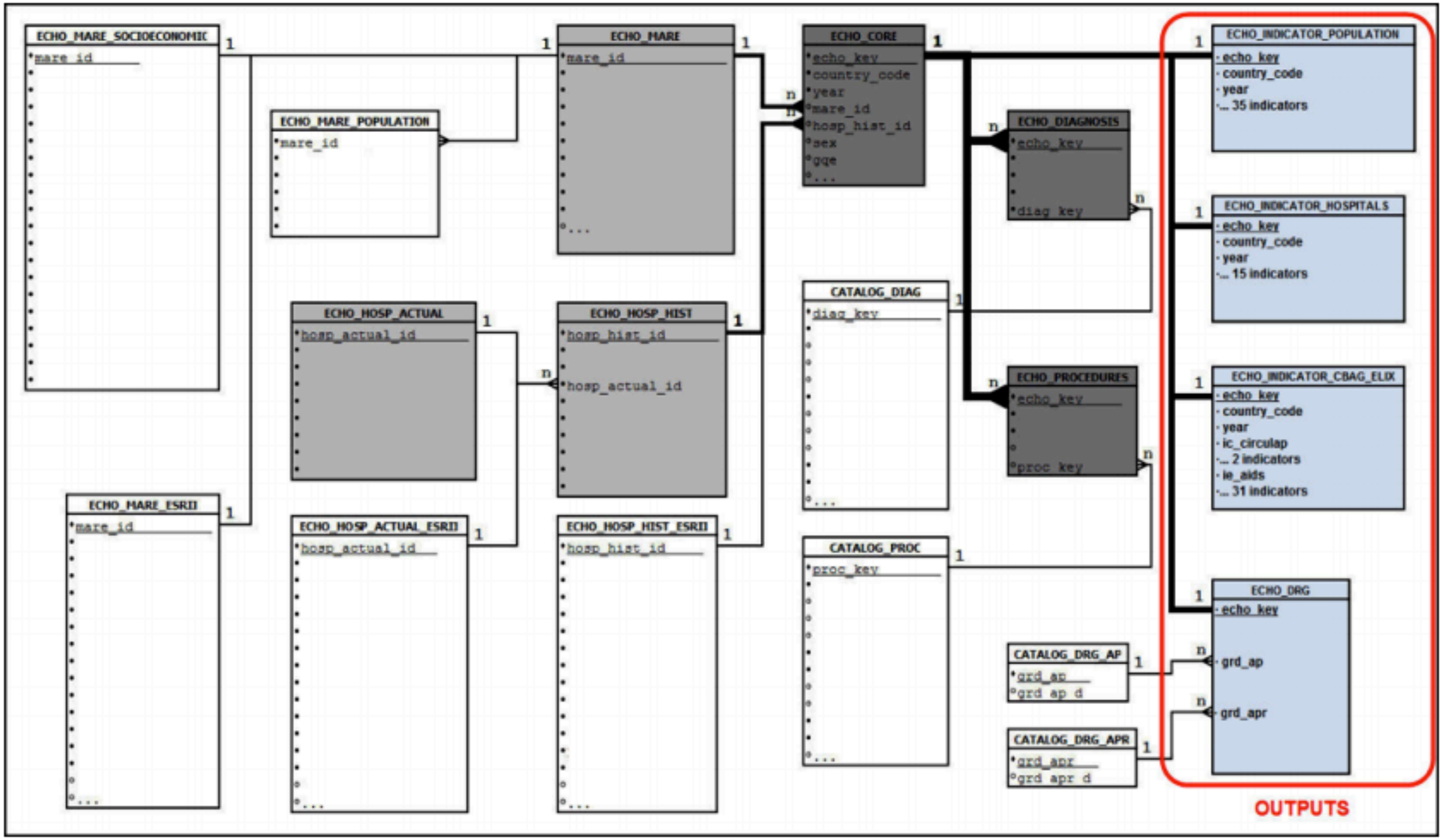

Note: White boxes represent the different origins of data (hospitalizations, demographics, socioeconomic information and supply information); dark grey boxes are catalogues for the units of analysis (hospitals and geographic regions); black boxes represent the joint resulting data set and catalogues for diagnoses and procedures; pale grey boxes represent the final output, which are the performance indicators and modifiers. Connection lines: univocal key identifiers (_id) allow 1 to 1 or 1 to N linkage
